# Association of Macrophage Migration Inhibitory Factor (MIF) with Therapy Response and Clinical Outcomes in HPV-Related Head and Neck Squamous Cell Carcinoma: A Preliminary Report

**DOI:** 10.3390/curroncol33050265

**Published:** 2026-05-01

**Authors:** Janki Naidugari, Shruti Wadhwa, Benjamin Xie, Sarah Taheri, Indraneel Kulkarni, Luke Johnson, Heehwa G. Son, John Strickley, Shadmehr Demehri, Joongho J. Joh, Robert Mitchell, Rebecca Redman

**Affiliations:** 1 Department of Medicine, University of Louisville School of Medicine, Louisville, KY 40202, USA; 2 Brown Cancer Center, University of Louisville School of Medicine, Louisville, KY 40202, USA; 3 Center for Cancer Immunology, Center for Cancer Research, Massachusetts General Hospital and Harvard Medical School, Boston, MA 02114, USA; 4 Cutaneous Biology Research Center, Department of Dermatology, Massachusetts General Hospital and Harvard Medical School, Boston, MA 02114, USA; 5 Department of Dermatology, West Virginia University, Morgantown, WV 26506, USA; 6 Division of Dermatology, University of Louisville School of Medicine, Louisville, KY 40202, USA; 7 Department of Surgery, Division of Immunotherapy, University of Louisville School of Medicine, Louisville, KY 40202, USA

**Keywords:** MIF, HPV, biomarker

## Abstract

**Background:** Macrophage migration inhibitory factor (MIF) is a critical modulator of the tumor immune microenvironment (TME). Its clinical significance in head and neck squamous cell carcinoma (HNSCC) remains controversial because of HPV-dependent tumor biology and the limitations of single-timepoint biomarker assessments. This preliminary study evaluates whether dynamic changes in circulating MIF (ΔMIF) in an HPV-stratified longitudinal cohort reflect disease severity and treatment response. **Methods:** Ninety-six serial serum samples were analyzed from 27 HNSCC patients (22 HPV-positive, 5 HPV-negative) from diagnosis through therapy and follow-up. Serum MIF and anti-HPV16 E7 IgG were quantified by ELISA, and ΔMIF was defined as the change in MIF concentration between consecutive visits. **Results:** Baseline MIF did not correlate with clinical stage in the total cohort (*p* = 0.63). However, 56% of HPV-positive patients exhibited a positive correlation between elevated MIF and advanced stage. Following chemoradiotherapy, the HPV-negative group showed a consistent and significant decline in MIF (mean ΔMIF = −1.23, *p* = 0.031), corresponding with no evidence of disease (NED). In contrast, the HPV-positive group showed heterogeneous trajectories (mean ΔMIF = +0.21, *p* = 0.94), with several patients demonstrating paradoxical declines in MIF during active disease or relapse, followed by recovery upon reaching NED. In select cases, MIF dynamics were closely synchronized with anti-E7 IgG levels. **Conclusions:** Serum MIF dynamics are strongly dependent on HPV status. While MIF serves as a reliable therapy-monitoring marker in HPV-negative HNSCC, it may play a complex and paradoxical immunomodulatory role in HPV-positive disease. These preliminary findings support the need for larger prospective, HPV-stratified trials.

## Simple Summary

Head and neck cancers are categorized into two distinct types based on the presence of the human papillomavirus. While the protein known as macrophage migration inhibitory factor is recognized for promoting tumor growth and suppressing the immune system, its role as a marker for tracking disease progression has been unclear. This study monitored changes in this protein’s levels over time in patients undergoing treatment. The findings reveal that in virus-negative patients, the protein levels consistently drop after successful treatment, making it a reliable tool for monitoring recovery. However, in virus-positive patients, the protein levels fluctuate unpredictably and may even decrease during cancer relapse, suggesting a much more complex role in the immune response. These results highlight that doctors must consider a patient’s virus status when using immune markers to monitor cancer, potentially leading to more personalized follow-up care and better-informed clinical decisions.

## 1. Introduction

Head and neck squamous cell carcinoma (HNSCC) is now recognized as two biologically distinct clinical entities defined by human papillomavirus (HPV) status [1,2,3]. HPV-positive [HPV(+)] HNSCC, characterized by viral oncoproteins E6 and E7, exhibits an improved prognosis and enhanced sensitivity to concurrent chemoradiotherapy (CRT) compared with traditional tobacco-associated HPV-negative [HPV(−)] disease [4,5]. Despite these biological differences, current monitoring strategies rely primarily on standardized imaging and static biomarkers, which may fail to capture real-time shifts in the tumor immune microenvironment (TME) [6,7].

Macrophage migration inhibitory factor (MIF) is a pleiotropic pro-inflammatory cytokine recognized as a driver of tumor progression and immune evasion [8,9,10,11,12,13]. Within the TME, MIF promotes the recruitment of myeloid-derived suppressor cells (MDSCs) and suppresses cytotoxic T-cell activation [14,15]. However, the existing literature on MIF in HNSCC remains contradictory. While several studies report higher circulating MIF levels in HPV(−) patients, others suggest that HPV oncoproteins stimulate metabolic reprogramming that enhances MIF secretion [16,17,18,19,20,21].

These discrepancies highlight a critical limitation of prior studies: reliance on single-timepoint measurements. Because the TME is dynamic, static MIF levels at diagnosis cannot reflect treatment-induced immune remodeling or early signals of relapse. This preliminary report utilizes longitudinal serum MIF analysis (ΔMIF) to address this gap. By tracking patients from diagnosis through treatment and remission, we aim to determine whether MIF dynamics serve as an HPV-specific indicator of clinical outcomes.

## 2. Methods

### 2.1. Study Cohort and Data Collection

This study was approved by the University of Louisville Institutional Review Board (IRB #08.0388, #15.0582) [22]. Anonymized serum samples and clinical data were obtained from 27 HNSCC patients through the Brown Cancer Center Biorepository. Given the preliminary nature of this report, the cohort was selected based on the availability of at least two consecutive serum samples. Ninety-six total samples (2–6 per patient) were analyzed, spanning from initial diagnosis through follow-up visits. All specimens were processed using additive-free vacuum tubes, allowed to clot for 30 min, centrifuged, aliquoted, and stored at −80 °C, in accordance with standard serum biomarker handling protocols [22,23,24].

### 2.2. HPV Stratification and Classification

Patients were categorized into HPV(+) (*n* = 22, 18 analyzable) and HPV(−) (*n* = 5) groups using p16 immunohistochemistry (IHC), HPV DNA testing, and anti-E7 IgG serology [22,23]. p16 IHC is a validated surrogate marker for transcriptionally active HPV in oropharyngeal cancers [21,22,23,24,25,26]. The limited HPV(−) cohort is acknowledged as a constraint. When p16 or DNA results were unavailable, HPV status was confirmed by detection of circulating anti-HPV16 E7 antibodies.

### 2.3. Assay Protocols for MIF and Anti-E7 IgG

Serum MIF was quantified using a sandwich ELISA method (Quantikine Human MIF ELISA kit, R&D Systems, Minneapolis, MN, USA). Samples were measured in duplicate, and absorbance was read at 450 nm (with 540 nm correction) using a Synergy HT (BioTek Instruments, Winooski, VT, USA) instrument; the average of the duplicates was used for the final concentration [27,28]. To monitor viral-specific immune responses, anti-E7 IgG levels were measured using ELISA plates coated with bacterially expressed HPV16 E7 oncoprotein. Signals were detected using an alkaline phosphatase-conjugated secondary antibody, and data from the 30 min interval—providing the clearest signal differentiation—was utilized for analysis [22,23].

### 2.4. Statistical Analysis Framework

The association between baseline MIF levels and diagnostic stage was assessed using Spearman’s rank correlation coefficient, given the ordinal nature of staging data and the non-normal distribution of cytokine levels. For longitudinal analysis, the difference in MIF concentration between consecutive visits (ΔMIF) was compared using the Wilcoxon signed-rank test [6,20,22,27].

## 3. Results

### 3.1. Patient Characteristics and HPV Status

The cohort had a mean age of 60 years and consisted of 19 Caucasian males, 4 Caucasian females, and 4 African American males, with tumors predominantly arising in the tonsil and oropharynx (Table 1). All HPV(–) patients presented with advanced disease (Stage III/IV), whereas only 60.9% of the HPV(+) group were diagnosed at these stages. Although the majority of patients achieved no evidence of disease (NED) following cisplatin-based chemoradiotherapy (CRT), all documented cases of metastasis or relapse (Patients 1, 20, and 25) occurred exclusively within the HPV(+) subgroup.

### 3.2. HPV-Specific Correlation with Disease Severity

**Figure 1:**
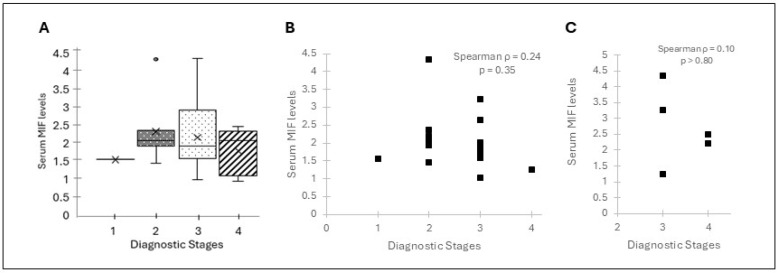
Association of baseline macrophage migration inhibitory factor (MIF) levels with diagnostic disease stage: (**A**) Distribution of baseline MIF levels across the entire patient cohort. A total of 27 HNSCC patients were evaluated for disease severity (Stage I–IV) at their initial clinical visit. Plasma MIF concentrations (ng/mL) were quantified via ELISA. Data are presented as box-and-whisker plots by clinical stage; boxes indicate medians and interquartile ranges. Statistical analysis was performed using the non-parametric Kruskal–Wallis test (*p* = 0.63). (**B**) Relationship between baseline MIF concentration and disease stage in HPV-positive HNSCC patients (*n* = 18). Serum MIF concentrations obtained at the first clinical visit were plotted against tumor stage. Spearman’s rank correlation analysis demonstrated a weak, non-significant positive association between baseline MIF and diagnostic stage (r = 0.24, *p* = 0.35). (**C**) Relationship between baseline MIF concentration and disease stage in HPV-negative HNSCC patients (*n* = 5). No meaningful correlation was observed between baseline MIF concentration and tumor stage in the HPV-negative subgroup (Spearman r = 0.10, *p* > 0.80). Each marker represents an individual patient.

At baseline, circulating MIF levels were broadly distributed across the cohort. No significant correlation was observed between baseline MIF and TNM stage in the overall population (Figure 1A; Kruskal–Wallis *p* = 0.63). However, HPV stratification revealed distinct biological patterns. Among HPV(+) patients, there was a weak positive correlation between baseline MIF levels and diagnostic stage, although this did not reach statistical significance (Figure 1B; Spearman r = 0.24, *p* = 0.35). In HPV(–) patients, no meaningful association was observed (Figure 1C; Spearman r = 0.10, *p* > 0.80). These results suggest that while MIF levels may tend to align with tumor stage in HPV(+) disease, a larger cohort is necessary to confirm the statistical strength of this relationship.

Although base-of-tongue tumors exhibited a modest downward trend in MIF dynamics (mean ΔMIF = −0.66), no statistically significant correlations were identified between primary tumor location and overall disease resolution.

### 3.3. Therapy Response and Post-Treatment MIF Trajectories

**Figure 2:**
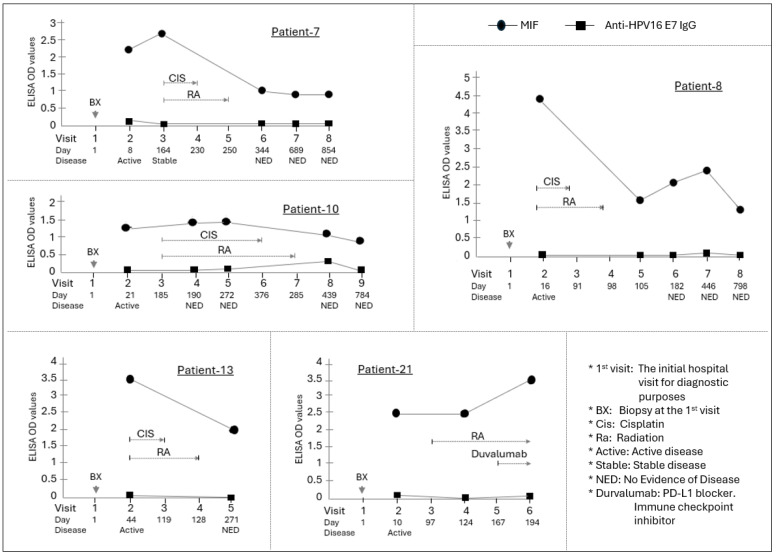
Longitudinal assessment of MIF levels in HPV(−) OPSCC patients. The HPV-negative cohort (N = 5) was defined by lack of p16 expression and/or negative HPV DNA testing. Critically, all patients in this group were diagnosed with advanced disease (Stage III or IV HNSCC) and received concurrent cisplatin and radiation therapy as their primary treatment. MIF concentrations (measured in ELISA) were evaluated at multiple critical time points. The line graph illustrates the individual change in MIF levels for each HPV-negative patient (N = 5, Patients 7, 8, 10, 13, and 21) across these stages. Individual graphs show the time course of MIF (circle) and anti-HPV16 E7 IgG (square) ELISA OD values for these patients. The *X*-axis indicates the visit number and the day relative to the initial hospital visit (Day 1). Below the *X*-axis, the disease status at each visit is indicated. The primary chemoradiotherapy (CIS/RA) course is shown with brackets above the data points. Arrows indicate specific clinical procedures or events. A significant reduction in MIF levels was observed post-treatment compared to the diagnosis time point, with an average ΔMIF of −1.23 (*p* = 0.031, Wilcoxon matched-pairs signed-rank test). The dotted line represents the mean MIF level at each time point. Expression of anti-HPV16 E7 IgG was examined in these patients. Four of the patients remained negative throughout the entire process. However, one patient (Patient 10) showed a low-level increase in the antibody 15 months after the initial diagnosis, though the concentration remained very low.

Cisplatin-based CRT provided the clearest stratification of MIF dynamics by HPV status. In the HPV(−) cohort, MIF levels declined significantly following treatment (mean ΔMIF = −1.23, *p* = 0.031), closely corresponding with achievement of NED (Figure 2).

In contrast, the HPV(+) group displayed markedly heterogeneous trajectories (mean ΔMIF = +0.21, *p* = 0.94). A categorical summary of these diverse longitudinal MIF patterns across the entire cohort—contrasting the uniform response in HPV(−) patients with the heterogeneity in the HPV(+) group—is presented in Table 2. Notably, 36% of HPV(+) patients maintained or increased MIF levels despite clinical remission. Even among HPV(+) patients receiving intensified triple-agent therapy (cisplatin, paclitaxel, and carboplatin), MIF levels remained largely unchanged, indicating distinct biological regulation of MIF pathways compared with HPV(−) disease.

### 3.4. Disease Outcomes, MIF Dynamics, and Anti-E7 IgG

**Figure 3:**
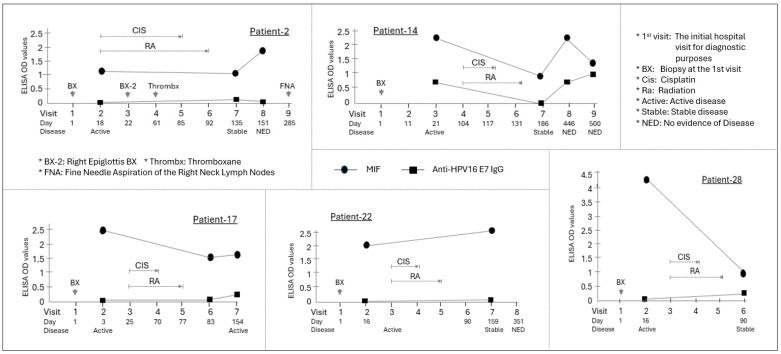
Longitudinal assessment of MIF levels in five HPV(+) OPSCC patients who developed active or stable disease within six months of primary chemoradiotherapy. Among the patients confirmed to be HPV(+) by p16 expression and HPV DNA testing, five patients (Patients 2, 14, 17, 22, and 28) showed active or stable disease within six months of chemoradiotherapy. The average change in MIF (DeltaMIF) for these patients was −1.55. Individual graphs show the time course of MIF (circle) and anti-HPV16 E7 IgG (square) ELISA OD values for five patients. The *X*-axis indicates the visit number and the day relative to the initial hospital visit (Day 1). Below the *X*-axis, the disease status at each visit is indicated. The primary chemoradiotherapy (CIS/RA) course is shown with brackets above the data points. Arrows indicate specific clinical procedures or events. Compared to the two patients (Patients 2 and 22) whose MIF levels did not change significantly, the remaining three patients (60%) experienced a decrease in MIF levels during the period of active disease following the initial chemoradiotherapy. Two of these patients (Patients 2 and 14) showed an increase in MIF levels to or above the initial diagnostic levels at the moment of disease clearance. Notably, Patient 14 was the sole case in which the MIF expression pattern closely resembled the anti-E7 IgG pattern across all four measurements.

The overall ΔMIF differed significantly between the two groups (*p* = 0.009), with the HPV(−) cohort showing a mean decrease of −1.62 and the HPV(+) cohort showing a mean increase of +0.06. Paradoxically, 80% of HPV(+) patients with active disease exhibited lower MIF levels during the disease phase compared with their baseline or NED state (Figure 3).

**Figure 4:**
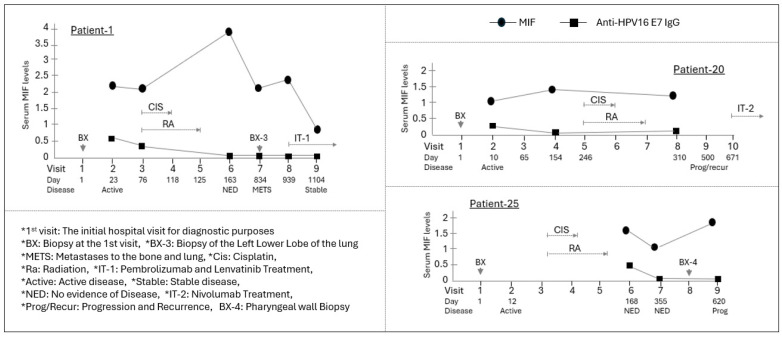
Longitudinal assessment of MIF levels in HPV(+) OPSCC patients who experienced disease relapse. Three HPV(+) patients (Patients 1, 20, and 25) experienced disease relapse following the clearance of their primary disease. While sufficient results could not be obtained for Patient 20 and Patient 25 due to a lack of sample availability throughout the entire course, Patient 1 was studied with samples secured across the entire course, from the primary disease to metastasis and the subsequent treatment of the secondary disease. Individual graphs show the time course of serum MIF levels (circle) and anti-HPV16 E7 IgG (square) for three HPV-positive patients. The *X*-axis indicates the visit number and the day relative to the initial hospital visit (Day 1). Below the *X*-axis, the disease status at each visit is indicated. The primary chemoradiotherapy (CIS/RA) course and subsequent treatments are shown with brackets and labels above the data points. Patient 1, who was diagnosed with distant metastatic lung cancer after treatment for the primary disease, received paclitaxel and carboplatin at 3.5 and 5.5 months, respectively, following the diagnosis of metastasis. Despite these therapeutic interventions, the patient continued to exhibit active disease three years after the initial presentation. This patient’s MIF level significantly increased after primary treatment, but at the time of relapse, approximately 23 months later, the MIF level was observed to have decreased to a value comparable to that at the initial diagnosis of the primary tumor. Subsequently, a reduction in the MIF level was noted following the administration of immunotherapy.

In Patients 2 and 14, MIF levels declined during active disease but recovered to baseline upon achieving NED. Relapse cases (Patients 1 and 25) demonstrated particularly complex patterns; for example, Patient 1 showed a spike in MIF at NED followed by a sharp decline during metastatic relapse (Figure 4).

Finally, Patient 14 exhibited strong synchronization between MIF and anti-E7 IgG levels, with both markers decreasing during active disease and recovering upon return to NED, suggesting a coordinated relationship between viral immune responses and MIF dynamics.

## 4. Discussion

Our longitudinal findings suggest that MIF dynamics in HNSCC are highly dependent on HPV status, underscoring distinct biological behaviors between virally driven and non-viral tumors. We explicitly acknowledge the small sample size (*n* = 27) and the limited HPV(−) cohort (*n* = 5) as significant constraints. Consequently, while our Spearman correlation analysis between baseline MIF and tumor stage did not reach statistical significance, the observed trends provide preliminary evidence for HPV-specific biological patterns. These data should be interpreted as preliminary observations intended for hypothesis generation rather than definitive clinical guidelines.

The consistent decline in MIF in HPV(−) patients post-treatment supports its role as a traditional tumor-associated and immunosuppressive factor in non-viral HNSCC. MIF has been implicated in promoting tumor growth and immunosuppression in HNSCC, including the recruitment of myeloid-derived suppressor cells (MDSCs) and suppression of cytotoxic immune infiltration [13,14,15,16,17,18]. As cisplatin-based chemoradiotherapy reduces tumor mass and associated immunosuppressive cell populations [29,30], circulating MIF levels decline. Despite the small sample size, the significant decrease in MIF within the HPV(−) cohort (*p* = 0.031) suggests that serial MIF monitoring is a potentially valuable non-invasive tool for tracking therapeutic responses in HPV(−) disease.

In contrast, the heterogeneous trajectories and lack of a significant post-treatment decline in the HPV(+) group (*p* = 0.94, Wilcoxon signed-rank test) suggest a more complex immunomodulatory role. As summarized in Table 2, the high variability in response patterns within the HPV(+) cohort contrasts sharply with the consistent decline observed in the HPV(−) group. HPV(+) tumors harbor viral antigens that can stimulate adaptive immune responses, contributing to a more immune-inflamed tumor microenvironment relative to HPV(−) counterparts [3,4,5]. Under certain inflammatory conditions, MIF can support T-cell activation and effector functions, indicating context-dependent roles beyond classical immunosuppression [16,17,18,19]. In HPV(+) HNSCC, a decrease in MIF during active disease or relapse may reflect immune escape or collapse of anti-tumor immune surveillance rather than clinical improvement.

Thus, a single low MIF value in an HPV(+) patient should not be prematurely interpreted as remission without corroborating clinical and radiographic evidence. Emerging data suggest that in virally driven cancers, MIF may participate in shaping immune activation status, with diminishing levels potentially signifying suppressed immune activity during tumor progression.

In conclusion, our findings indicate that serum MIF dynamics offer potential utility for HPV-stratified monitoring in HNSCC: a decline in MIF corresponds to treatment response in HPV(−) disease, whereas the more variable and paradoxical patterns in HPV(+) cases may reflect underlying immune modulation. Larger prospective trials are essential to validate these observations and to elucidate the interplay between MIF and the tumor microenvironment, including MDSCs, CD8^+^ T cells, and immune surveillance in HPV-associated tumor contexts.

## Figures and Tables

**Table 1:** Demographic, clinical, and HPV status in head and neck squamous cell carcinoma patients.

Patient #	Age	Race/ Gender	Stage	Tumor Location	P16 IHC	HPV DNA	Anti-HPV16 E7 IgG	HPV Group	1st * Outcome	Relapse
1	51	W/M	2	T	POS	POS	POS	(+)	NED	METs
2	60	W/M	4	S	POS	NR	NEG	(+)	NED	
3	78	W/F	3	FM	NR	POS	NEG	(+)	NED	
4	54	W/M	1	L	POS	NR	POS	(+)	NED	
5	64	W/M	3	T	POS	POS	NEG	(+)	NED	
6	62	W/M	3	T	POS	NR	NEG	(+)	NED	
7	62	W/F	4	O	NEG	NR	NEG	(−)	NED	
8	65	B/M	3	BT	NEG	NR	NEG	(−)	NED	
9	54	W/M	2	T	POS	POS	NEG	(+)	NED	
10	48	W/M	3	T	NR	NEG	NEG	(−)	NED	
11	43	B/M	3	T	POS	NR	POS	(+)	NED	
13	55	W/F	3	O	NEG	NR	NEG	(−)	NED	
14	55	W/M	2	T	NEG	NR	POS	(+)	Stable ^a^	
15	37	W/M	2	O	POS	NR	POS	(+)	NED	
16	58	W/M	3	BT	POS	NR	NEG	(+)	NED	
17	61	W/M	2	BT	POS	NR	NEG	(+)	Stable ^b^	
18	51	W/M	4	T	POS	NR	NEG	(+)	NED	
19	60	B/M	3	T	POS	NR	NEG	(+)	NED	
20	63	W/M	3	BT	POS	NR	POS	(+)	IND	PRG
21	88	W/M	4	T	NEG	NR	NEG	(−)	IND	
22	65	W/M	2	LO	POS	NR	NEG	(+)	Stable ^c^	
23	55	W/F	3	T	POS	NR	POS	(+)	NED	
24	54	W/M	-	L	POS	NR	POS	(+)	NED	
25	47	W/M	3	T	POS	POS	POS	(+)	NED	PRG
26	49	B/M	4	T	POS	NR	NEG	(+)	NED	
27	57	W/M	3	T	POS	NR	NEG	(+)	NED	
28	70	W/M	2	TP	POS	POS	NEG	(+)	Stable ^d^	

Oropharynx: T = tonsil, O = oropharynx (unspecified), BT = base of tongue, LO = lateral wall of OP, TP = tonsillar pillars. Other: S = supraglottis (larynx), FM = floor of mouth (oral cavity), L = lymph nodes (unknown primary). POS = positive; NEG = negative; NR = no results; NED = no evidence of disease; IND = indeterminate; PRG = progression. 1st *: within 60 days of completing the first treatment (Tx). Stable ^a^: Outcome 55 days after completing the first treatment, but the outcome changed to NED at Day 315 post-Tx. Stable ^b^: Outcome 71 days after completing the first treatment. Stable ^c^: Outcome 25 days after completing the first treatment. Stable ^d^: Outcome 69 days after completing the first treatment, but the outcome changed to NED at Day 192 post-Tx.

**Table 2:** Summary of longitudinal MIF trajectory patterns.

Trajectory Pattern	HPV(+) (*n* = 22)	HPV(−) (*n* = 5)
Concordant decline with NED (MIF drops as disease clears)	7	4
Increase with progression/relapse (MIF rises with tumor)	4	1
Paradoxical decline (MIF drops during active disease/relapse)	2	0
No clear pattern/Fluctuating	9	0
Total	22	5

## Data Availability

The original contributions presented in this study are included in the article. Further inquiries can be directed to the corresponding author.

## Abbreviations

The following abbreviations are used in this manuscript:

HPV	Human Papillomavirus
T	Tonsil
O	Oropharynx (unspecified)
BT	Base of Tongue
LO	Lateral Wall of Oropharynx
Tp	Tonsillar Pillars
S	Supraglottis (Larynx)
FM	Floor of Mouth (Oral Cavity)
L	Lymph Nodes (Unknown Primary)
POS	Positive
NEG	Negative
NR	No Results
NED	No Evidence of Disease
IND	Indeterminate
PRG	Progression
